# Quality of life as assessed by adults with cerebral palsy

**DOI:** 10.1371/journal.pone.0191960

**Published:** 2018-02-05

**Authors:** Alba Maestro-Gonzalez, M. Cruz Bilbao-Leon, David Zuazua-Rico, Jose M. Fernandez-Carreira, Ricardo F. Baldonedo-Cernuda, M. Pilar Mosteiro-Diaz

**Affiliations:** 1 Department of Medicine, Nursing Area, University of Oviedo, Oviedo, Spain; 2 Hospital Universitario Central de Asturias, Oviedo, Spain; 3 Department of Education Science, University of Burgos, Burgos, Spain; 4 Health Service of Principality of Asturias. Hospital Público Comarcal de Jarrio, Jarrio, Spain; University Children's Hospital Tuebingen, GERMANY

## Abstract

**Aim:**

We explored the quality of life of adults with cerebral palsy without an intellectual disability and the predictors of quality of life.

**Background:**

Because cerebral palsy is a disease that manifests in childhood, much of the research into quality of life for those dealing with it focuses on children; there are few studies that evaluate the quality of life of adults with cerebral palsy. Therefore, it is important to consider their perceptions in order to improve their general wellbeing and self-determination.

**Design:**

This was a descriptive, cross-sectional study.

**Method:**

Quality of life was measured using the GENCAT Quality of Life Scale. Demographic and personal variables were also collected and examined. Participants comprised 75 adults (58.7 percent men, mean age = 40.84 years) with cerebral palsy who were members of the National Cerebral Palsy Association of Spain between 2014 and 2015. A linear multivariate model was examined as well.

**Results:**

The overall mean score indicator of participants’ quality of life was 103.29, which corresponds to the 56.6th percentile on the GENCAT scale. Examining the level of qualification, we found significant differences in the factors “personal development” and “self-determination,” and those with a university education obtained higher scores than their less-educated counterparts. Having a partner was related to higher quality of life standard scores. After constructing a linear model, it was observed that maintaining sexual relationships was another factor that increased participants’ quality of life.

**Conclusion:**

This study highlights the importance of social and romantic relationships to achieve a better quality of life in adults with cerebral palsy who do not have an intellectual disability. Social integration and sexuality education programs should be developed to improve their quality of life.

## Introduction

The concept of cerebral palsy (CP) has evolved over time [1–3]; it is currently defined as “a group of disorders of the development of movement and posture, causing activity limitation, that are attributed to non-progressive disturbances that occurred in the developing fetal or infant brain. The motor disorders of cerebral palsy are often accompanied by disturbances of sensation, cognition, communication, perception, and/or behavior, and/or by a seizure disorder” [4]. The evolution of this concept has played a critical role in changing the disability paradigm, which has developed from a “deficit theory” to a “biopsychosocial conception,” supported by cultural importance and the participation of disabled people [5]. Moreover, CP is the most common physical impairment in childhood. Its prevalence in developed countries is estimated at 2–2.5 cases per 1,000 live births [6, 7]. However, the proportion of school-aged children in the United States diagnosed with CP is 3–4 per 1,000 people. This disorder affects more than 17 million people in the world and 25 percent of the affected children will never be able to walk. Just half of those with a severe intellectual disability survive to become adults [8].

The concept of Quality of Life (QoL) is relatively new in health evaluation. It is difficult to define the concept because it is not a simple notion. Although there is as yet no consensus on defining criteria, most of the international scientific community accepts that it includes the fulfillment of eight basic needs that represent the essence in everyone’s existence: emotional, economic, and physical well-being, interpersonal relationships, personal development, self-determination, social inclusion, and rights [9]. Several studies have affirmed that some factors such as interpersonal relationships [10], sexuality [11], and physical conditions [12] are also crucial to a higher QoL.

For disabled persons, gaining self-determination is paramount as it implies a willingness to take control of one’s own opportunities, decisions, and environmental conditions [13, 14]. This is vital because it promotes individuals to be active participants in the search for solutions to their needs. However, interest in self-determination is very recent, so few established theoretical models exist. Furthermore, comprehension of the concept and the consequences of QoL are still expanding [3]. Wehmeyer is the most recognized author on this subject, and his definition is the most accepted and well-known: “Self-determination is the process by which a person’s action is the primary causal agent in one’s life and to make choices regarding one’s actions free from undue external influence or interference” [15]. Therefore, discussing self-determination refers to rights, skills, actions, ethics, responsibility, authority, and freedom. It relates to the abilities and attitudes required to act as the primary causal agent in one’s life and to achieve adequate social interdependence [16]. This concept is of particular interest if we consider that, until recently, disabled people tended to be treated as if they were passive, depending on their families, professionals, and society in general to make their life decisions [17, 18]. However, this trend is changing; it is now recognized that disabled individuals should be actively involved in obtaining all the services and materials they need [5, 19].

Despite some progress having been made, the participation of disabled people locally, regionally, or nationally in the agencies where their life decisions are made is still low; even associations for persons with disabilities generally have administrations that are led by people without those disabilities. It is unknown whether this problem exists because disabled people do not have the qualifications to fulfill these positions or because people’s expectations of disabled individuals are too low. Either way, the qualifications of disabled people are an important factor on the path to self-determination and promoting a better QoL [20, 21].

Although several studies evaluating the QoL of people with CP have been conducted focusing on young people or children [22–25], most of these subjects go on to become adults, and QoL for that population has received far less attention.

Therefore, in this study, we analyzed the QoL of adults with CP who did not have an intellectual disability to elucidate relevant sociodemographic factors and predictors.

## Materials and methods

### Design

This was a descriptive, cross-sectional study conducted in 2014 and 2015.

### Participants

For this study, we employed a purposive sample comprising adults with CP without intellectual disability (N = 93). General intellectual functioning was defined according to the Wechsler Adult Intelligence Scale (WAIS-IV), adapted to the Spanish population, as individuals with a score of 70 or higher, according to Wechler's classification [26]. Inclusion criteria were the following: adults with CP without an intellectual disability, aged 18 years or older, belonging to one of two specific Spanish communities that are part of the National CP Association. Participants provided written, informed consent. Overall, 75 people answered the questionnaire (response rate = 80.64 percent).

### Procedure

Cerebral Palsy Spanish Association and Asturias’ Research Ethics Committee (n° 112/17) approved this specific study. The questionnaire introduction explained the main objective of the study, assured privacy and confidentiality, and briefly described how to correctly complete the questionnaire and informed consent form. A data collection sheet and the GENCAT scale were self-administered by all participants, as we considered it necessary that participants should complete this information independently. Doing so ensured a higher level of accuracy and truthfulness, and simultaneously promoted their rights and freedom of expression as individuals. The instructions to complete the form were provided by the same professional (instructed in evaluation instruments) who was present during the collection period.

### Data collection

#### Instruments

The QoL GENCAT scale was used to collect the data [27]. This scale was designed for use as a measurement to aid in the continuous improvement of social services. It is a validated scale that is accepted nationally and internationally [27]. GENCAT is based on Schalock and Verdugo’s [28] multidimensional model, which reflects individual desires in relation to eight essential needs: emotional wellbeing, interpersonal relations, material wellbeing, personal development, physical wellbeing, self-determination, social inclusion, and rights. The scale evaluates QoL through 69 items, all expressed in the third person in declarative format, and were answered using a 4-point Likert scale (“*never or hardly ever*,” “*sometimes*,” “*frequently*,” and “*always or almost always*”).

In the GENCAT Scale, different scales according to the type of the studied population exist: general population scale, elderly population scale (aged 50 years and older), disabled people with an intellectual disability scale, and a scale for other groups (e.g., drug addicts, physical disability, and mental health problems). We used the “physical disability and mental health problems” scale.

Furthermore, we evaluated the factors and life conditions that, according to research, have some impact on QoL [10, 12, 29]. We designed an item bank with sociodemographic data (e.g., age, sex, and the Gross Motor Function Classification System), environmental conditions (e.g., place of residence, adapted home, and qualifications), and intimate aspects related to social interaction (e.g., people with whom they live, conjugal life, and sexual relationships).

Sociodemographic data was initially gathered using 28 questions. All were shown to 10 professionals (two each of psychologists, special educators, social educators, teachers, pedagogues, and nurses) to evaluate their meaning. Once their contributions were collected, only items that had an agreement average above 75 percent were included in the questionnaire. This resulted in 10 final items: age, gender, Gross Motor Function Classification System score, place of residence, adapted home, people with whom they live, qualifications, partnership, active sexual relationship and first sexual relationship. The item “adapted home” refers to installing devices in one’s home that make daily life easier. The item “Gross Motor Function Classification System” [30, 31] is a five-level classification system that describes the gross motor function of children/youth with CP in terms of self-initiated movement with particular emphasis on sitting, walking, and wheeled mobility. Those classified as “Level I” can generally walk without restrictions. Those classified as “Level V” are generally very limited in their ability to move themselves around even with the use of assistive technology.

#### Data analysis

The obtained data were computationally processed using SPSS 20.0 for Windows PC (licensed to the University of Oviedo). To describe quantitative variables, an arithmetic mean was used as a measure of central tendency and standard deviation (SD) and rank were used to measure data dispersion. Total frequencies and percentages were used to describe qualitative variables. The qualitative variables were compared using Pearson’s chi square test. To analyze quantitative variables among two groups, the student t-test for independent samples was applied. If a qualitative variable had more than two categories, an ANOVA was carried out after verification of the normality hypothesis (Kolmogorov Smirnov Test) and a Tukey post-hoc test was performed when the differences were statistically significant. To study the linear relation among quantitative variables, a Pearson correlation was calculated.

Finally, a linear multivariate model was designed. The level of statistical significance applied through the whole study was set at p < .05.

#### Ethical considerations

This study was approved by the ethics committee of the National CP Association and Asturias’ Research Ethics Committee (n° 112/17).

## Results

Participants’ characteristics are presented in Table 1. There was a significant relationship between age and education level (*p* = .001).

**Table 1:** Participant characteristics (N = 75).

		Mean (Minimum/Maximum)	SD
Age		40.84 (19/69)	11.75
		N	%
Participants by age group	20 years old or younger	3	4.0
	21–30 years old	11	14.7
	31–40 years old	25	33.3
	41–50 years old	19	25.3
	51–60 years old	12	16.0
	61 years old or older	4	5.3
Gross Motor Function Classification System	I	2	2.66
	II	9	12
	III	15	20
	IV	13	17.3
	V	36	48
Sex	Male	44	58.7
	Female	31	41.3
Place of residence	Personal accommodation	10	13.5
	Family home	42	56.8
	Association	22	29.7
Adapted home	Yes	40	54.1
	No	34	45.9
People with whom they live	First-degree relative	37	52.9
	Second-degree relative	3	4.3
	Partner	6	8.6
	Association companions	21	30.0
Qualifications	No studies	31	41.9
	Primary studies	17	23.0
	Secondary studies	10	13.5
	Professional qualifications	12	16.2
	Higher studies	4	5.4
Partnership	Yes	20	28.2
	No	51	71.8
Active sexual relationship	Yes	17	24.3
	No	53	75.7
First sexual relationship	Yes	28	40.0
	No	42	60.0

### GENCAT QoL scale

It is important to note that the overall mean score indicator of the participants’ QoL was 103.29, which corresponds to the 56.6th percentile on the GENCAT scale (Cronbach’s alpha = 0.92). The items with the lowest mean score were “has a satisfactory sexual life” (mean = 1.87 points, SD = 1.24, range = 1–4) and “presents with depression symptoms” (mean = 1.95 points, SD = 1.08, range = 1–4). Those with higher mean scores were “has good personal hygiene” (mean = 3.89 points, SD = .39, range = 1–4) and “reports feeling loved by the most important people to him or her” (mean = 3.81 points, SD = .54, range = 1–4).

When analyzing the eight factors that compose QoL and relating them to the different sociodemographic variables we found the following results. A relationship between age and “personal development” was identified. This factor was increased in the youngest participants. It was observed that people who lived in their own residence had a higher level of self-determination than those who lived in a center or group home. However, those who lived with family or at a center obtained higher scores in the “social inclusion” factor than those who lived in their own residence. In addition, those who had their homes adapted obtained higher scores for the “physical wellbeing” factor. Participants who lived with their partner obtained a higher score in the factors “interpersonal relationships,” “personal development,” and “self-determination.” Examining the level of education, we found significant differences in the factors “personal development” and “self-determination.” Moreover, those who currently had a partner obtained higher scores in “interpersonal relationships,” “personal development,” “rights,” and the global QoL index. Finally, participants who maintained sexual relationships had higher scores in “interpersonal relationships,” “personal development,” “self-determination,” and the global QoL index (Tables 2 and 3). However, no differences were found for the “material wellbeing” or “emotional wellbeing” factors.

**Table 2:** Comparing the GENCAT Quality of Life Scale (part 1)*.

Factor	Variable		Mean	p
Interpersonal relationships	People with whom they live	First-degree relative	30.73	.038
		Second-degree relative	31.33	
		Partner	35.00	
		Association companions	28.71	
	Partnership	Yes	33.90	.004
		No	29.22	
	Active sexual relationships	Yes	33.76	.002
		No	29.19	
Personal development	Age (years)	≤20	27.33	.049
		21–30	24.09	
		31–40	26.12	
		41–50	24.63	
		51–60	21.83	
		>60	25.25	
	People with whom they live	First-degree relative	24.78	.018
		Second-degree relative	25.00	
		Partner	28.67	
		Association companions	24.14	
	Qualifications	No studies	23.58	.017
		Primary studies	25.24	
		Secondary studies	26.00	
		Professional qualifications	25.08	
		Higher studies	28.75	
	Partnership	Yes	27.00	< .001
		No	24.29	
	Active sexual relationship	Yes	26.24	.039
		No	24.23	
Physical wellbeing	Adapted home	Yes	26.63	.047
		No	24.94	
	Partnership	Yes	25.15	.009
		No	26.51	

*Only significant results are shown.

**Table 3:** Comparing the GENCAT Quality of Life Scale (part 2)*.

Factor	Variable		Mean	p
Self-determination	Place of residence	Personal accommodation	27.20	.010
		Family home	26.14	
		Association	22.14	
	People with whom they live	First-degree relative	25.22	.019
		Second-degree relative	28.33	
		Partner	31.00	
		Association companions	22.24	
	Qualifications	No studies	22.03	.006
		Primary studies	26.47	
		Secondary studies	27.89	
		Professional qualifications	27.64	
		Higher studies	30.25	
	Partnership	Yes	29.40	< .001
		No	23.36	
	Active sexual relationships	Yes	29.12	< .001
		No	23.42	
Social inclusion	Place of residence	Personal accommodation	22.60	.041
		Family home	26.29	
		Association	24.09	
Rights	Qualifications	No studies	32.03	.025
		Primary studies	36.41	
		Secondary studies	36.00	
		Professional qualifications	35.50	
		Higher studies	32.00	
	Partnership	Yes	36.30	.011
		No	33.53	
Global Quality of Life Index	Partnership	Yes	226.56	.007
		No	213.47	
	Active sexual relationships	Yes	231.07	.030
		No	213.25	
	People with whom they live	First-degree relative	221.06	.047
		Second-degree relative	229.33	
		Partner	230.20	
		Association companions	208.67	

*Only significant results are shown.

Post-hoc subgroup analyses, presented in Table 4, showed significant differences between the following groups and variables:

**Table 4:** Post-hoc subgroup analyses*.

Factor	Variable	Comparison Groups	Mean	p
Interpersonal relationships:	People with whom they live:	Partner	35.00	.019
		Association companions	28.71	
Personal development:	age	31–40	26.12	.006
		51–60	21.83	
	People with whom they live	Partner	28.67	.038
		First-degree relative	24.78	
		Partner	28.67	.018
		Association companions	24.14	
	Qualifications	No studies	23.58	.045
		Higher studies	28.75	
Self-determination	Place of residence	Personal accommodation	27.20	.030
		Association	22.14	
		Family home	26.14	.011
		Association	22.14	
	People with whom they live	Partner	31.00	.003
		Association companions	22.24	
	Qualifications	No studies	22.03	.019
		Secondary studies	27.89	
		No studies	22.03	.019
		Professional qualifications	27.64	
		No studies	22.03	.021
		Higher studies	30.25	

*Only significant results are shown.

To study the possible relationship between sociodemographic variables and the GENCAT scale, we developed a linear model. For this, we included variables that showed p-values lower than .20 derived from the previous analysis (i.e., active sexual relationships, partnership, education, and people with whom they live) and the variables age and sex. We determined that the “maintenance of sexual relationships” was related to the GENCAT and increased QoL (Table 5).

**Table 5:** Linear model.

Model	Coefficient	Sig.
Active sexual relationships		
(Intercept)	101.56	< .001
Active sexual relationships: yes	10.286	.030

Adjusted R-squared = 0.05914; analysis of variance p-value = .03 < .05.

## Discussion

The aim of this study was to analyze the QoL of adults with CP who did not have an intellectual disability and investigate possible predictors of a higher QoL.

The overall mean score indicator of participants’ QoL was 103.29, which corresponds to the 56.6th percentile on the GENCAT scale. This means adults with CP tend to perceive their QoL the same way the general population does. Consistent with this finding, Hergenröder and Blank [10] stated that subjective wellbeing and general satisfaction were not diminished in adults with CP. However, the sample population in Morgan et al. [32] reported that they perceived their health-related QoL to be poor.

We agree with Davis et al. [23], who affirmed in their study of adolescents with CP and their parents that self-determination is a crucial factor, especially when teenagers opined about their future and felt good about themselves. Similarly, Tarsuslu and Livanelioglu [12] stated that while physical aspects such as mobility and pain are stronger indicators of health-related QoL in healthy populations, for young adults with CP, psychological factors such as communication skills and social inclusion are more important. Moreover, we agree with Hergenröder and Blank [10], who stated that interpersonal relationships are critical to a higher QoL. It is also noteworthy that in Wiegerink et al.’s study [11], they found a relationship between age and time spent dating and stated that being of an older age was associated with greater sexual activity.

With respect to sociodemographic variables, we focused on middle-aged adults with CP (mean = 40.84 years). In other studies, very little information about this age range exists. Most publications were centered on children and adolescents [29, 33]. Some similar studies have addressed middle-aged adults [10, 34]; however, these were largely male-dominated, which was also the case in Young et al. [29].

Regarding place of residence, we found it striking that most participants lived in their family home; however, almost half had not had their home adapted. Comparing our results with other studies that investigated the places where adults with CP reside, Blum et al. [35] showed similar results, affirming that 90 percent lived in parental households, 7 percent lived in group situations, and only two people lived alone. However, that study addressed adolescents who are typically not independent. Andersson and Mattson [34] concluded that 84 percent lived in their own house, with or without home help service.

Regarding participants’ educational level, the data showed a declining percentage and an inverse proportional relationship with age. The percentages of our study are different from those obtained by Hergenröder and Blank [10], who concluded that 32 percent did not have professional qualifications, 44 percent did, and 24 percent were working towards higher education. In addition, in Van der Dussen et al.'s study [36], 53 percent had completed secondary education, 15 percent had not, and 12.5 percent attended a school for people with special needs. In other words, in our sample, participants had less education (5.4 percent had completed higher education, 16.2 percent had professional qualifications, 13.5 percent had completed secondary education, 23 percent had completed primary studies, and 41.9 percent had no educational record). Our relatively higher proportion rate in level 5 of the GMFCS might explain this fact.

Regarding personal relationships, consistent with other studies, 71.8 percent reported that they were not currently involved in a sexual relationship. In Van der Dussen et al. [36], 87.5 percent were single, 12.5 percent were married or living with their partner, and 5 percent had children. Wiegerink et al. [37, 38] showed that 23 percent of people in their sample were in a relationship. On the other hand, in a survey about sexuality [39], single men with CP displayed lower scores on a standardized sexuality instrument concerning sexual information, experience, attitude, psychological symptoms, affection, and satisfaction than did healthy single men. Moreover, both middle-aged men and women with CP had higher scores for psychological items; however, women reported a lower rate of sexual satisfaction [39]. Finally, it is worth highlighting the relationship found in the linear model, where the maintenance of sexual relationships was related to the GENCAT and increased their QoL. No studies were found relating these two factors, and therefore it would also be interesting to take them into account for future researches.

### Limitations

The results of this study must be interpreted within the context of the sample. These findings cannot be extrapolated to other conditions due to the specificity of the sample. Although the participation rate could be considered high, there is a possible overrepresentation of adults classified as level 5 in GMFCS. Further studies involving a larger sample size and including other countries and cultures need to be conducted to strengthen these conclusions. Moreover, it is possible that some participants did not feel comfortable discussing relationships and sexuality with a female researcher (in a few cases, the main researcher had to fill in questionnaires for those people with writing disabilities). Furthermore, it could be interesting to analyze CP classifications and possible differences depending on the type or distribution [40]. However, despite these limitations, we confirmed our hypotheses and our results can serve as a basis for future investigations about the QoL of adults with CP.

## Conclusion

The QoL conceptual framework created by Schalock and Verdugo [28] was a useful guide for this study. The results indicate that adults with CP perceive their QoL as equivalent to that of healthy adults. “Interpersonal relationships,” “personal development,” and “social inclusion” were the most important factors for QoL, whereas “material wellbeing” and “physical wellbeing” were the least. Our data also support the theory that a high QoL is related to having a current sexual relationship. These results have social implications for nursing practice. It is important to encourage sexual education among adults with CP and adapt existing social inclusion programs to increase their QoL.
